# Ovarian Tumor of Unusual Size—Successful Removal

**Published:** 1892-09

**Authors:** Charles H. Richmond

**Affiliations:** Livonia, N. Y.


### OVARIAN TUMOR OF UNUSUAL SIZE—SUCCESSFUL REMOVAL.

BY CHARLES H. RICHMOND, M. D., Livonia, N. Y.

I DO not often report operations, but this case, while not unique, is unusual in these times, and presents an opportunity for the study of the differential signs in such cases.

The case was that of Mrs. B., of Groveland, N. Y., a woman 54 years old, tall and naturally large, but now emaciated. Has two grown-up children. She measured five feet three inches in circumference at the umbilicus, the enlargement being prominent and pendulous. The ribs were distended, fluctuation was marked over every part of the abdomen, flatness on percussion was observed all over in all positions, the finger in the vagina felt no pouching downward, but could, by a quick movement, communicate a vibration to the hand over the abdominal walls, the uterus was apparently normal in size and movable, and there was no hardening of the pelvic roof. Her feet were somewhat swollen, one limb, which had once been the seat of "milk leg," being considerably enlarged and presenting a small-sized ulcer. Her bowels were habitually constipated. Her countenance did not show marked organic disease likely to produce ascites, nor was the ovarian countenance well marked.

The only history that could be obtained was that enlargement of the abdomen was first noticed nearly seven years previous to my examination, and that she was tapped about five years previous, about twenty pounds of a light straw-colored serum, so far as recollected, having been removed. When the abdomen refilled, the fulness seemed to be even, no bunch being noticed.

I had never seen a case of fluid accumulation of any sort which approached this in size. Chronic peritonitis was excluded by mobility of the uterus and the absence of hardening of the pelvic roof, so that the diagnosis rested between the ovarian cyst and ascites. The spreading of the lower ribs, together with prominence of the belly, without much flattening while the patient was lying on the back, seemed to favor the existence of tumor. It seemed also that if the fluid were free in the abdominal cavity, that quantity would cause bulging into the pelvis. She had been treated for ascites, but always by irregular practitioners.

I advised an exploratory operation with a view to ovariotomy if a tumor were found, because it seemed more probable that a cyst existed, and a diagnosis would most likely be as difficult with the fluid withdrawn, especially if there was much adhesion. Moreover, the delay of a radical operation incident to the tapping might prove disastrous at that stage of the growth, provided it proved to be cystic. Accordingly, on June 3, 1892, assisted by Dr. Nellie V. Chappell, of Buffalo, (then of South Lima,) Dr. McGowan, of Conesus Center, and Dr. Jones, of Scottsburgh, I proceeded to operate after the usual antiseptic preparations. The exploratory incision was about two inches in length. The diagnosis of cyst was soon made, when the incision was lengthened about an inch, the cyst tapped and removed in the usual way. The cyst wall was extensively adhered to the omentum and parietes, so that a portion of the former was cut away, and separation from the abdominal wall, in a considerable portion, could only be accomplished by dissecting off the fibrous coat with the knife. Several daughter cysts were formed, each containing a different kind of fluid. In one the fluid seemed very oily. That contained in the large cyst was light colored, much resembling the fluid of ascites. The entire operation occupied about two hours, on account of the time taken to draw away the fluid and to separate the adhesions. The weight of the cysts and contents was fully eighty pounds.

The cavity left was appalling, for I feared that all the air could not be excluded from beneath the diaphragm, and that septicemia might result from the extensive raw surfaces. The patient took the anesthetic (chloroform, followed by ether,) well, and sustained no apparent shock. She was left in charge of Dr. Jones. The temperature never went above or 101½°. It was ordinarily below 100°. She took no opiates during the first few days. The stitches were removed after eight days without suppuration. Persistent vomiting took place after thirty-six hours, which was attributed to errors in diet on account of misunderstanding. This was followed by a severe diarrhea, a source of much trouble for two or three weeks. Small mural abscesses developed in the wound, so that complete union was delayed, making it just seven weeks before the patient was on her feet, although she was substantially well before that time. The puckered condition of the abdominal walls and the sunken aspect of the wound made it more liable to become infected by foreign matter. The scar is about one and a half inches in length. The peritoneum seems well united.

